# Phenotypic characterization of NK cells in 5-year-old children exposed to maternal HIV and antiretroviral therapy in early-life

**DOI:** 10.1186/s12865-024-00674-4

**Published:** 2024-12-19

**Authors:** Hope Mataramvura, Julia Jӓger, Ana Jordan-Paiz, Lovemore Ronald Mazengera, Felicity Zvanyadza Gumbo, Madeleine J. Bunders, Kerina Duri

**Affiliations:** 1 Immunology Unit, Department of Laboratory Diagnostic and Investigative Sciences, Faculty of Medicine and Health Sciences, University of Zimbabwe, UZ-FMHS), Harare, Zimbabwe; 2 Department of Virus Immunology, Leibniz Institute of Virology, Hamburg, Germany; 3 Paediatrics and Child Health Unit, UZ-FMHS, Harare, Zimbabwe; 4 III. Department of Medicine, University Medical Centre Hamburg-Eppendorf, Hamburg, Germany

**Keywords:** Children, Early-life maternal HIV exposure, Preconception/post-conception antiretroviral therapy exposures, NK cell inhibitory markers, CD56^bright^ and CD56^dim^, Perforin and granzyme B, Morbidity in a low resource setting

## Abstract

**Background:**

HIV-exposed uninfected (HEU) children are at increased risk of morbidity during the first years of life. Although the immune responses of HEU infants in early-life are relatively well described, studies of natural killer (NK) cells in older HEU children are lacking. NK cell subsets were analysed in HEU children and compared to those in HIV unexposed uninfected (HUU) children aged ~ five years.

**Methods:**

Multi-parametric flow cytometry was used to characterize peripheral blood-derived NK cell CD56, CD16, CD57, NKG2A and KIR3DL1/KIR2DL2/L3 expression, including intracellular perforin and granzyme B. NK cell subsets were compared between HEU children exposed to prenatal antiretroviral therapy (ART) from conception [long-term (HEULT)]; those exposed to ART during pregnancy [medium-term (HEUMT)] with continued exposure throughout the breastfeeding period and HUU peers. Furthermore, clinical data of the children, including sick clinic visits and hospitalizations documented in morbidity diaries from birth to 5 years were compared between HEU and HUU groups. Frequencies of CD56^bright^ and CD56^dim^ NK cell were correlated with these clinical parameters.

**Results:**

139 children were enrolled however, 133 comprising 43 HEULT, 38 HEUMT and 52 HUU were included in the main analyses. Total NK cell, CD56^bright^ nor CD56^dim^ NK cell proportions differed between HEU and HUU children. However, HEULT children had lower frequencies of CD56^dim^ NK cells compared to HEUMT children, (*p* = 0.002) which maintained significance after controlling for preterm birth, *p* = 0.012. No differences were observed between HEULT and HUU. The expressions of NKG2A, KIR3DL1/KIR2DL2/L3 and CD57 on CD56^bright^ and CD56^dim^ NK cells were similar between the three groups. Furthermore, the frequencies of granzyme B and perforin double positive NK cells were similar between the HUU with HEULT and HEUMT children. CD56^dim^ NK cell counts had a significant moderate negative correlation with recurrent respiratory infections (rho=-0.38; *p* = 0.010) in HUU children and negatively correlated with total sick clinic visits in HEUMT (rho=-0.40, *p* = 0.064).

**Conclusion:**

The proportions of total NK cell, CD56^bright^ and CD56^dim^ NK cells, NK cells inhibitory and differentiation surface marker expression and cytolytic granule-positive cells were similar between HEU and HUU children. These data suggest that early-life HIV/ART exposure may not result in major changes in NK cell subsets at 5 years of age.

**Supplementary Information:**

The online version contains supplementary material available at 10.1186/s12865-024-00674-4.

## Introduction

According to UNAIDS more than 85% of pregnant women living with HIV now have access to antiretroviral therapy (ART) for their health and the prevention of mother-to-child transmission (PMTCT) of HIV [1]. Universal access to lifelong ART in pregnant women living with HIV has resulted in a continued increase in the population of infants exposed to HIV/ART *in utero* and throughout the breastfeeding period. HIV-exposed uninfected (HEU) children populations was approximately 14.8 million globally in 2018 [2].

Although HEU children are not infected, they have higher rates of morbidity compared to HIV-unexposed uninfected (HUU) children [3]. HEU children have been noted to present with increased risk of sick clinic visits and hospitalizations due to mostly respiratory tract infections and diarrhoeal diseases within the first two years of life [4–7]. Most studies have focused on analyses of children in the first 2 years of life and, there is a paucity of data on the outlook in relatively older HEU children [8, 9].

An altered immune system has been suggested to contribute to increased morbidity in HEU infants younger than 2 years [10, 11]. In infants, innate immunity plays a crucial role in non-specific responses to infection early in life as the adaptive immune system matures. Natural killer (NK) cells are innate lymphocytes whose main subsets are CD56^bright^ and CD56^dim^CD16^+^ cells, which make up approximately 10% and 90% of peripheral NK cells, respectively [12, 13]. The CD56^bright^ NK cells mainly produce cytokines such as tumor necrosis factor (TNF) and interferon-γ, while CD56^dim^ NK cells display cytolytic activities through the secretion of perforin and granzymes [14]. Natural killer cell anti-viral functions and maturation is regulated through expression of inhibitory or activating receptors on the cell membrane [15, 16], such as Killer Immunoglobulin-like Receptors (KIRs) 3DL1, 2DL2/L3 and NKG2A, which mostly bind to HLA class I ligands and non-classical HLA molecules [17]. The inhibitory receptors NKG2A and KIRs are critical for the education of NK cells and their subsequent functionality [12].

Maternal factors including HIV disease severity, chronic immune activation and inflammation in women living with HIV have been shown to impact the health outcomes of their infants [10, 18]. A proinflammatory environment and cell stress due to HIV infection can affect the maternal-fetal interface, which may skew the fetal immune system and have consequences for postnatal immune competence [19]. The timing of maternal ART initiation has been shown to further impact the health outcomes of HEU. Goetghebuer and colleagues showed that the reduction of maternal antibody transfer, and increased immune cell activation were more pronounced in children born to mothers who initiated ART post-conception than in those born to mothers who initiated ART preconception [20]. Antiretroviral therapy significantly improves the quality of life of people living with HIV (PLWH) however, it has also been associated with adverse effects in children and adults [21, 22]. Furthermore, ART can cross the placenta, specifically nucleoside reverse transcriptase inhibitors have been detected at high concentrations in cord blood [23] raising concerns about the potential long term effect of early maternal ART exposure during this vulnerable phase of development.

To date, most studies in HEU children have demonstrated altered proportions, phenotypes and effector functions of NK cells in infants under one year of age [12, 13]. However, it is unclear how these phenotypes change upon weaning and whether they persist at older ages. Therefore, we aimed to characterize NK cells subsets in HEU children in comparison with those in their HUU peers at approximately five years of life.

## Materials and methods

### Study design and participants

This investigation was a nested study under the University of Zimbabwe Birth cohort study (UZBCS). Children aged 4-5.5 years were enrolled between May and July 2022.

Briefly the UZBCS enrolled pregnant women of at least 20 weeks gestation from high-density areas in Harare 2016–2019 and conducts clinical follow ups of the mother-child dyads from delivery, weeks 1, 6, 10, 14, 24, 36, 48, 72 and 96 and once a year to date [24]. By design, half of the pregnant women enrolled into the study were living with HIV and 99.3% of them were on *Teno*fovir, *Lam*ivudine and *E*favirenz (TENOLAM-E) therapy at enrolment for PMTCT and for their own health. Mothers were encouraged to exclusively breastfeed during the first 6 months of life. In line with the national guidelines, all HEU children were given Nevirapine and Cotrimoxazole prophylaxis until weaning or testing positive for HIV DNA by PCR, whichever occurred first as previously described [24]. Due to lifelong universal access to ART and effective monitoring, the number of vertical transmissions has decreased resulting in few infected children participating in the UZBCS [25]. Therefore, these studies focused on HEU children.

At delivery mothers were issued study morbidity diaries for the documentation of symptoms, clinical diagnoses and treatment of any illness in the child. To assess childhood morbidity in the cohort, we analysed existing clinical records from the morbidity diaries to obtain the documented sick clinic visits, the clinical diagnosis at each visit and any hospitalization resulting from the illness for all the children in this study from birth to 5 years.

The duration (days) of *in utero* ART exposure was categorized as maternal ART initiation preconception (long) or ART initiation post-conception up to 4 weeks before birth (medium) term as previously described [26].

### Ethical considerations

Ethical approval for this study was obtained from the Joint Research Ethics Committee for University of Zimbabwe and Parirenyatwa Group Hospitals: JREC (JREC/81/20) and Medical Research Council of Zimbabwe: MRCZ (MRCZ/A/2662). The mother/guardian of each child provided written informed consent and all the women were literate.

### Blood collection and assays

Venous whole blood was collected in EDTA tubes. Since all the children were above 18 months of age, the HIV Rapid Test Kit Determine™ HIV-1/2 (Abbott-Diagnostics, USA) was used to confirm the HIV status of exposed children. The viral load test was performed using HIV RT-PCR (Roche, USA) at the Sally Mugabe National Microbiology Reference Laboratory. Full blood counts were performed on a Mindray Haematology BC3600 Analyser (Shenzhen, China).

### Peripheral blood mononuclear cells isolation

Peripheral blood mononuclear cells (PBMCs) were isolated within four hours of blood collection using density gradient centrifugation of blood loaded on Ficoll medium (Capricorn). The cells were resuspended in Roswell Park Memorial Institute (RPMI) 1640 (MP Biomedicals) supplemented with 10% heat-inactivated foetal bovine serum (FBS) (Capricorn) and used for subsequent flow cytometry analyses.

### Flow cytometry

PBMCs were incubated in fluorescence-activated cell sorting (FACS) buffer, which was made from commercial Hank’s Balanced Salt Solution (HBSS) (MP Biomolecules) supplemented with 0.5% FBS containing optimally titrated antibody concentrations. The antibodies included the following: cluster of differentiation (CD) 3/CD14/CD19-AF700 (BioLegend catalogue numbers; #300424, #301822, #302226 respectively). The NK surface markers used were CD16-BV785 (BioLegend, #302046), CD56-BV510 (BioLegend, #318340), CD57-PEDazzle594/BV605 (BioLegend, #359620), KIR3DL1-PerCP-Cy5.5 (BioLegend, #312718), KIR2DL2/L3-PerCP-Cy5.5 (BioLegend, #312614), NKG2A-PE-Cy7 (Beckman Coulter, #B10246) and LIVE/DEAD fixable near-IR dye (Thermo Fisher Scientific). The cells were inclubated for 20 min at 4 °C in the dark and then washed twice with FACS buffer.

Next the cells were permeabilized using Fixation/permeabilization solution (BD Biosciences) followed by washing with Perm/Wash buffer (BD Biosciences). The cells were incubated with Perm/Wash buffer containing optimal concentrations of the antibodies against Perforin-BV711 (BioLegend, #308130) and Granzyme B-PE (BioLegend, #372208) for 20 min at 4^o^C in the dark. The samples were washed as previously indicated and then resuspended in FACS buffer. The data was acquired using a BD LSRFortessa flow cytometer (BD Biosciences). Fluorescence minus one (FMO) controls were used to set gates for the phenotypic markers (Supplementary Fig. 1). At least 100 000 events were acquired from each sample, and a minimum cell viability of 75% was considered for all samples to be included in the statistical analyses.

### Software and statistical analyses

A FACSDiva 8 (Becton Dickinson) was used for flow cytometry data acquisition. FlowJo 10.8.1 (FlowJo, LLC, Ashland OR, USA) software was used for gating the flow cytometry data. GraphPad Prism 9.0.0 (121) (GraphPad Software, La Jolla, CA, USA) and R programming version 4.1.0 were used for the statistical analyses. The Kruskal-Wallis test with Dunn’s correction for multiple comparisons was used to compare the 3 groups. Associations between categorical variables were determined using the Fisher’s exact test. The Spearman’s method with corrections for multiple testing was used for correlations of continuous variables. A *p* value < 0.05 was considered significant.

## Results

### Study population

A total of 139 children were enrolled however, 133 children were included in the main analyses; 43 HEULT, 38 HEUMT, 52 HUU of whom 48.9% were female. The other 6 children were HEI children and were excluded from the main analyses due to the small sample size. Three of the HEI children seroconverted within six weeks of birth while the other three seroconverted after 6 weeks from birth (Supplementary Table 1). Within the HEI group, five children were on treatment, three of whom maintained HIV viral suppression while the other two as well as one child not on treatment had viral loads > 1000 copies/ml at 5 years (Supplementary Table 2). The median viral load of the HEI children was 1525 copies/ml (IQR: 582.5-14700.0) at sample collection. Although most children were born at term, there was a trend towards increased risk (OR = 5.74; CI: 1.2-28.0, *p* = 0.033) of preterm birth in long term ART exposure compared to the unexposed children. The HEULT group had the highest proportion of premature children (19%) than the HEUMT (5.7%) and HUU (3.9%) groups (*p* = 0.05), (Table 1). The median duration on ART for the HEUMT mothers was 110 days (IQR: 64.5-184.5). Birth weight did not differ between the HEU and HUU groups.

**Table 1:** Maternal factors during pregnancy and infant birth outcomes for children stratified by HIV and ART exposure status

Characteristic Median (IQR) [*N*(%)]	HEU (*N* = 81)		HUU (*N* = 52)	*P* value^^^	*P* value^#^
	**HEU MT (** ***N*** ** = 38)**	HEU LT **(** ***N*** ** = 43)**			
**Maternal factors**					
**Age (years)**	30.0 (24.0-35.5)	33.0 (29.0-35.5)	29.0 (25.0–34.0)	0.078	0.118
**Viral load** (***n* = 81)**					
Suppressed	33 (86.8)	39 (95.1)	N/A	0.247	N/A
Unsuppressed	5 (13.2)	2 (4.9) (missing = 2)			
**CD4 count **(***n* = 81)**	347 (230–589)	422 (308–535)	N/A	0.384	N/A
**Birth outcomes**					
**Gestational age at birth (weeks)**					
Preterm (< 37)	2 (5.7)	8 (19.0)	2 (3.9)	0.050	0.126
Term (≥ 37)	33 (94.3) (missing = 3)	34 (81.0) (missing = 1)	50 (96.1)		
**Birth weight(grams)**					
<2500	6 (15.8)	4 (9.3)	3 (5.8)	0.304	0.369
≥2500	32 (84.2)	39 (90.7)	49 (94.2)		
**Sex**					
Female	19 (50.0)	23 (53.5)	23 (44.2)	0.726	0.721
Male	19 (50.0)	20 (46.5)	29 (55.8)		

^- *p* values for comparisons between HEULT, HEUMT and HUU, #- *p* values for comparisons between HEU and HUU. Abbreviations: HEU- HIV-exposed uninfected, HUU- HIV-unexposed uninfected, IQR- interquartile, LT- long term, MT- medium term

### Frequencies of CD56^bright^ NK cells and CD56^dim^ NK cells in HEU and HUU children

**Fig. 1:**
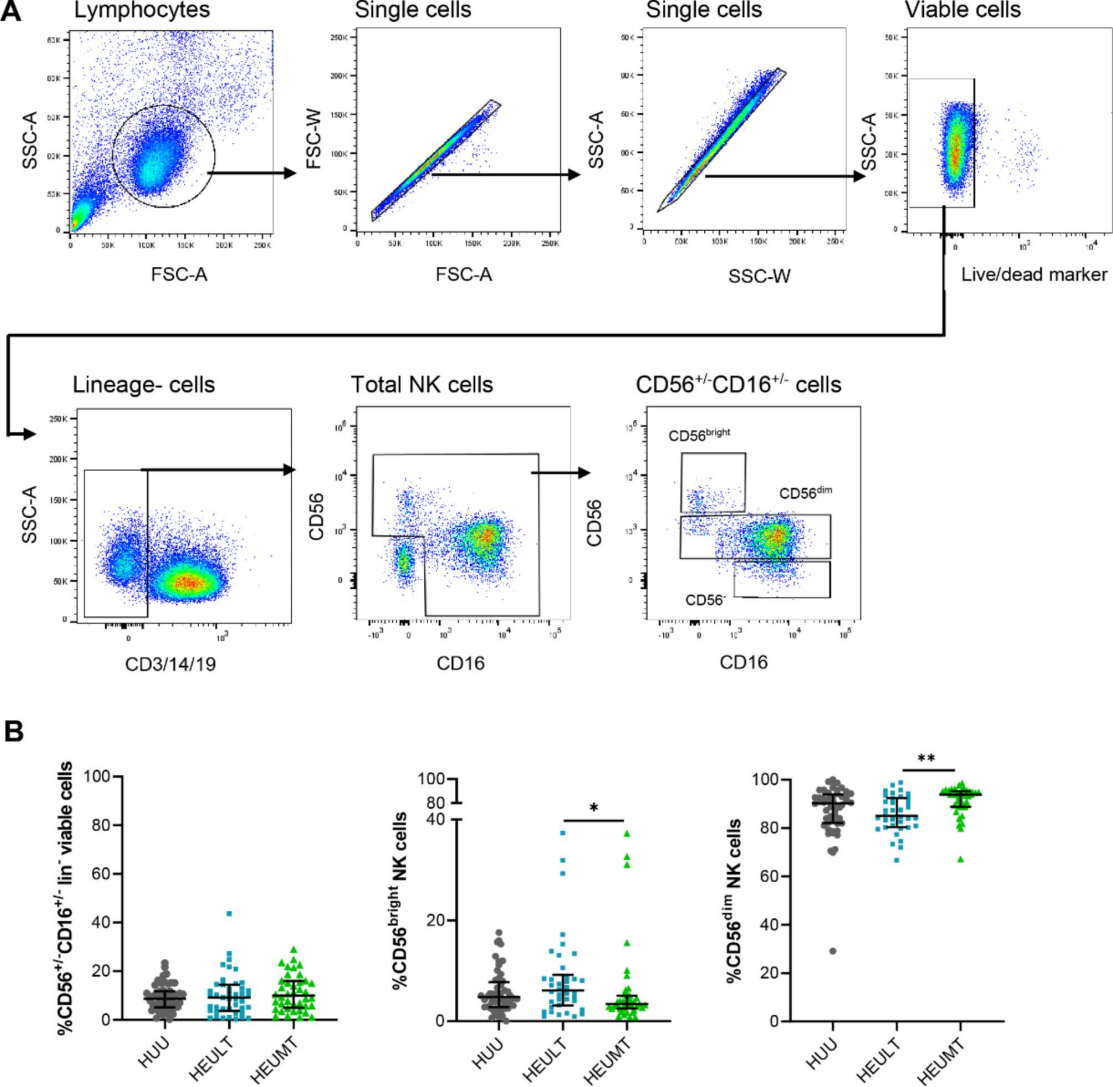
Gating strategy and NK cell frequencies in HUU, HEUMT and HEULT children. **A**: Gating strategy for selecting viable CD3-CD14-CD19- (lineage negative) single NK cells based on the expression of CD56 and CD16. **B**: The frequencies of total NK cells among viable peripheral blood lymphocytes and subsets (CD56^bright^ and CD56^dim^ NK cells). Only CD56^dim^ NK cell frequencies maintained significance after controlling for age and preterm birth. *=*p* < 0.01, **= *p* < 0.001. Abbreviations: HEU: HIV-exposed uninfected, HUU: HIV-unexposed uninfected, LT: long term, MT: medium term, NK: natural killer

NK cells and NK cell subsets were identified using the gating strategy shown in Fig. 1A. Total NK cells constituted approximately 8.9% of the total viable lymphocytes. The CD56^bright^ and CD56^dim^ subsets comprised 4.6% (IQR: 2.9–8.3) and 89.5% (IQR: 81.7–94.2) of the total NK cells, respectively (Fig. 1B). Before stratification by duration of ART exposure, HEU children had similar frequencies of CD56^bright^ NK cells (4.6%; IQR: 2.9–9.1) compared to HUU children (4.6%; IQR: 2.9–6.9), *p* = 0.478. The same was true for the CD56^dim^ NK cells in HEU and HUU groups (88.8%; IQR: 80.9–94.2 and 90.6%; IQR: 83.2–93.6 respectively), *p* = 0.453. However, after stratification the HEULT group had a higher median frequency of 6% (IQR: 3.0-9.1) CD56^bright^ NK cells compared to 3.4% (IQR: 2.5-5.0) in the HEUMT group, *p* = 0.025 . In addition, the HEULT had a significantly lower median frequency 85.0% (IQR: 80.5–92.4) of CD56^dim^ NK cells compared to 93.8% (IQR: 88.8–95.3) in HEUMT group, *p* = 0.002 (Fig. 1B**)**. After controlling for age and preterm birth, only the difference in CD56^dim^ NK cells between HEULT and HEUMT groups remained significant, *p* = 0.012. The CD56^negative^ NK cell subset was notably more distinguishable in HEI children compared to other groups. A comparison of the frequencies of CD56^negative^ NK cells showed that HEI children had significantly increased CD56^negative^ subset compared to HEULT, HEUMT and HUU groups although the analysis was underpowered (Supplementary Fig. 2). In summary, differences in NK cell populations between HEULT and HEUMT when compared to HUU children were not detected.

**Fig. 2:**
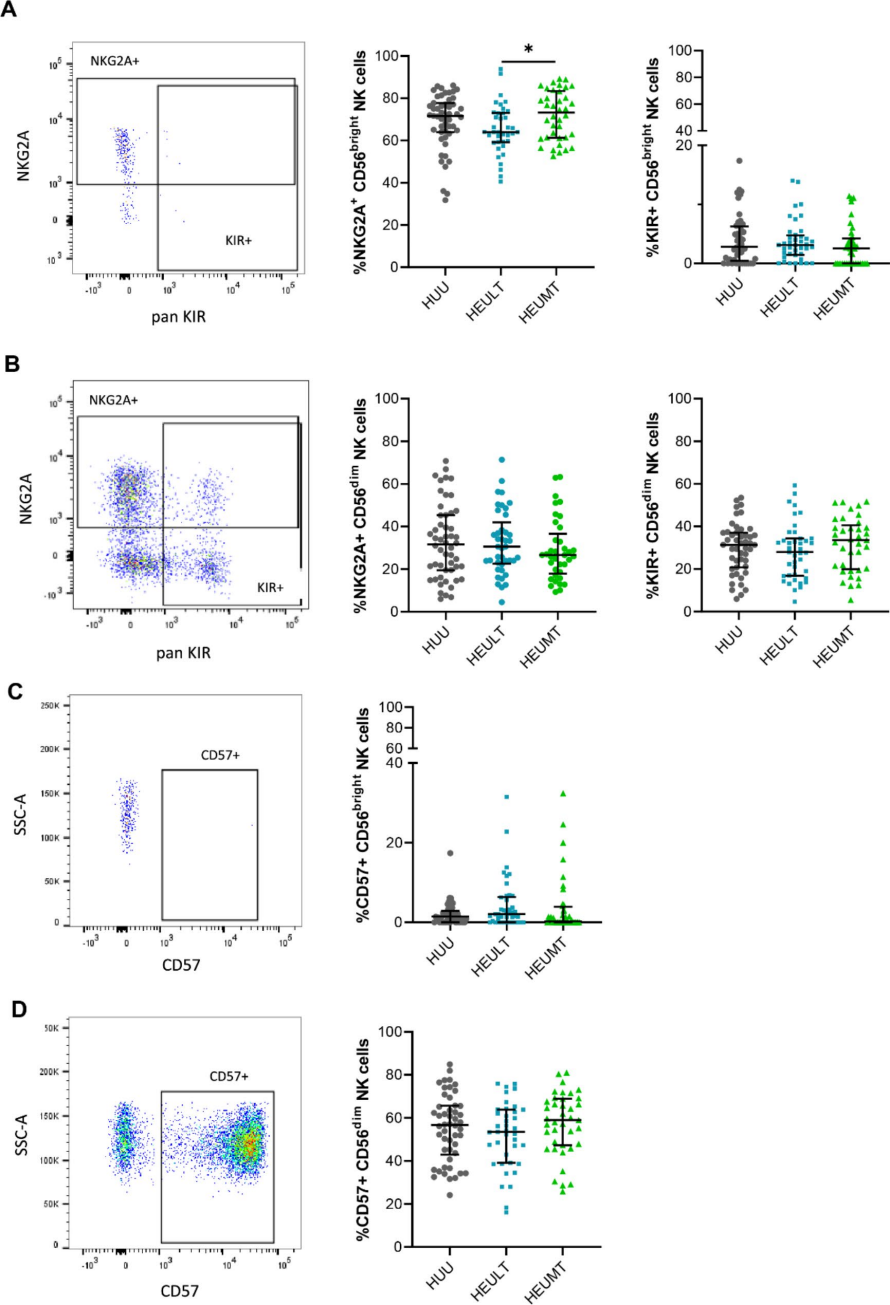
Representative flow plots and summarized data of the percentages of  **A** CD56^bright^ and **B** CD56^dim^ NK cells expressing KIR3DL1/KIR2DL2/L3 (pan-KIR) and NKG2A and **C** CD57 expression by CD56^bright^ NK cells and **D** by CD56^dim^ NK cells among HUU, HEULT and HEUMT children. *=*p* < 0.01. Abbreviations: HEU: HIV-exposed uninfected, HUU: HIV-unexposed uninfected, LT: long term, MT: medium term, NK: natural killer

### Inhibitory receptor expression on CD56^bright^ and CD56^dim^ NK cells in HEU and HUU children

The frequencies of CD56^bright^ and CD56^dim^ NK cell subsets expressing panKIR (KIR3DL1 and KIR2DL2/D3) were similar between HEU and HUU children and did not differ according to the duration of early-life ART exposure (Fig. 2A). The proportions of NKG2A^+^ CD56^bright^ NK cells tended to be lower in HEULT children than in HUU children but were significantly lower (*p* = 0.041) in HEULT children than in HEUMT children (Fig. 2A). Frequencies of NKG2A^+^CD56^dim^ NK cells were also similar between HUU, and both groups of HEU children (Fig. 2**B**). Furthermore, the percentages of CD57^+^ CD56^bright^ NK cells and CD56^dim^ NK cells did not differ among the groups (Fig. 2**C**, **D**). Taken together, NK subsets expressing CD57, KIR and NKG2A did not differ between HEU and HUU children.

### The frequencies of perforin and granzyme B positive NK cells were similar between HEU and HUU children

To assess the cytotoxic potential of NK cells intracellular perforin and granzyme B in NK cells were measured. As expected CD56^bright^ NK cells had very little to no intracellular perforin and granzyme B positive cells (Supplementary Fig. 3A). The frequencies of perforin^+^ granzyme B^+^ CD56^dim^ NK cells were similar among HEULT, HEUMT and HUU children (Supplementary Fig. 3B). Interestingly, the exploratory analysis of HEI children revealed lower frequencies of perforin and granzyme B double positive NK cells than in HUU children (Supplementary Fig. 4B). In summary, the comparable frequencies of perforin^+^ granzyme B^+^ CD56^dim^ NK cells in HEULT, HEUMT and HUU children indicate that maternal HIV nor prenatal ART exposure had a negative impact on the generation of perforin and granzyme B in CD56^dim^ NK cells.

### Childhood morbidity

As increased childhood morbidity has been observed in HEU children, childhood diaries recording diseases and kept by mothers were assessed. The main clinical diagnoses recorded were skin (27%), gastrointestinal (47%) and respiratory infections (59%) among the children. Approximately 56% of the hospitalizations occurred due to respiratory infections (data not shown). The analyses showed that sick clinic visits and hospitalizations at 6 weeks, 6 months, 12 months and beyond 12 months did not differ among the HEULT, HEUMT and HUU groups of children (Table 2). Although not the main objective of the study, unsurprisingly HEI children had the highest frequencies of sick clinic visits at all the four time points and of combined sick clinic visits from birth to 5 years although this difference did not reach significance (*p* = 0.518) (Supplementary Table 2). Taken together, analyses of morbidity diaries of children did not reveal increased morbidity in this cohort.

**Table 2:** Clinical and morbidity data for children stratified by HIV and ART exposure status at 5 years

Characteristic Median (IQR) [*N*(%)]	HEU (*N* = 81)		HUU (*N* = 52)	*P* value^^^	*P* value^#^
	**HEU MT (** ***N*** * = 38)*	HEU LT **(** ***N*** ** = 43)**			
**Children’s age at sample collection (years)**	5.0 (4.6–5.2)	5.2 (4.8-6.0)	5.2 (4.7-6.0)	0.086	0.139
**Clinical factors**					
Weight (kg)	17.7 (16.0-19.7)	17.9 (15.7–20.0)	17.7 (16.0-19.2)	0.822	0.585
MUAC (cm)	16.0 (15.4–17.0)	16.4 (15.5–17.0)	16.0 (15.5–17.0)	0.798	0.772
HB(g/dL)	13.5 (12.5–14.5)	13.4 (12.7–14.2)	13.4 (12.7–14.1)	0.668	0.403
WBC (10^9^ /L)	7.0 (5.5–8.9)	6.2 (5.2–7.1)	6.9 (5.7–8.6)	0.120	0.385
**Morbidity**					
Sick clinic visits at ≤ 6 weeks of age	1.0 (1.0–1.0)	1.0 (1.0–1.0)	1.0 (1.0–1.0)	0.549	0.273
Sick clinic visits at > 6 weeks-6months	1.0 (1.0–1.0)	1.0 (1.0–1.0)	1.0 (1.0–1.0)	0.855	0.601
Sick clinic visits at > 6 months-12months	1.0 (1.0–2.0)	1.0 (1.0-1.3)	1.0 (1.0–1.0)	0.596	0.399
Sick clinic visits beyond 12 months	1.0 (1.0–2.0)	1.0 (1.0–2.0)	1.0 (1.0–2.0)	0.974	0.865
Combined sick clinic visit (birth- 5 years)	2.0 (1.0–3.0)	2.0 (1.0–3.0)	2.0 (1.0–3.0)	0.398	0.222
Hospitalization					
Yes	3 (7.9)	4 (9.3)	6 (11.5)	0.875	0.560
No	35 (92.1)	39 (90.7)	46 (88.5)		

^- *p* values for comparisons between HEULT, HEUMT and HUU, #- *p* values for comparisons between HEU and HUU. Abbreviations: cm- centimetres, dL- decilitre, HB- haemoglobin, HEU- HIV-exposed uninfected, HUU- HIV-unexposed uninfected, IQR- interquartile, kg- kilograms, L- litre, LT- long term, MT- medium term, MUAC- mid upper arm circumference, WBC- white blood cell count

### NK cell subsets are associated with clinical parameters in HEU and HUU children

To assess whether NK cell subsets were correlated with childhood morbidity clinical parameters correlation analyses of clinical parameters with NK cell subsets in HEU and HUU children were performed. The proportion of CD56^dim^ NK cells had a weak negative correlation (rho=-0.26, *p* = 0.061) with recurrent respiratory infections in the total population of HEU and HUU children. According to stratified analyses of the individual groups, CD56^dim^ NK cells had a significant moderate negative correlation with recurrent respiratory infections (rho=-0.38; *p* = 0.010) in HUU children (Table 3). Within the HEUMT children CD56^dim^ NK cells had a moderate negative correlation with combined sick clinic visits and a weak correlation with recurrent respiratory infections (rho =-0.4, *p* = 0.064 and rho= -0.31, *p* = 0.104 respectively) (Table 3). In summary, lower proportions of CD56^dim^ NK cells were associated with increased childhood morbidity due to recurrent respiratory infections in the HUU but not in HEU children.

**Table 3:** Correlations of morbidity data with proportions of NK cell subsets among children stratified by HIV and ART exposure status

Clinical parameter rho (*p* value)	HEUMT (*n* = 38)	HEULT (*n* = 43)	HUU (*n* = 52)
**CD56**^**bright**^ **NK subset**			
6 months SCV	-0.200 (0.912)	-0.013 (0.869)	0.139 (0.730)
12 months SCV	-0.157 (0.838)	-0.420 (0.866)	-0.382 (0.358)
SCV beyond 12 months	0.260 (0.436)	-0.140 (0.596)	-0.200 (0.993)
5 years SCV	-0.300 (0.096)	0.027 (0.689)	-0.120 (0.325)
Overall SCV	0.320 (0.326)	-0.089 (0.812)	0.066 (0.493)
Recurrent respiratory infections	0.330 (0.110)	-0.032 (0.872)	0.160 (0.365)
Recurrent GIT	0.120 (0.559)	0.180 (0.384)	0.100 (0.770)
**Gran B + Per + CD56**^**bright**^ **NK subset**			
6 months SCV	-	-0.067 (0.875)	0.122 (0.821)
12 months SCV	-0.453 (0.551)	-0.078 (0.946)	0.400 (0.139)
SCV beyond 12 months	0.231 (0.838)	-0.130 (0.897)	0.260 (0.539)
5 years SCV	-0.115 (0.889)	-0.150 (0.447)	0.380 (0.254)
Overall SCV	-0.042 (0.961)	-0.220 (0.235)	0.130 (0.550)
Recurrent respiratory infections	0.151 (0.838)	-0.240 (0.456)	-0.081 (0.643)
Recurrent GIT	0.137 (0.853)	0.079 (0.985)	-0.150 (0.603)
**CD56**^**dim**^ **NK subset**			
6 months SCV	0.093 (0.912)	0.055 (0.851)	-0.019 (0.980)
12 months SCV	0.198 (0.798)	0.137 (0.869)	0.279 (0.493)
SCV beyond 12 months	-0.230 (0.512)	0.18 (0.490)	-0.002 (0.058)
5 years SCV	0.320 (0.080)	-0.065 (0.868)	0.140 (0.498)
Overall SCV	**-0.400 (0.0640)**	0.065 (0.786)	-0.150 (0.301)
Recurrent respiratory infections	**-**0.310 (0.104)	-0.080 (0.877)	**-0.380 (0.010)**
Recurrent GIT	-0.110 (0.502)	-0.140 (0.402)	-0.042 (0.980)
**Gran B + Per + CD56**^**dim**^ **NK subset**			
6 months SCV	-	0 (1.000)	0.087 (0.864)
12 months SCV	0.056 (0.960)	-0.077 (0.946)	0.135 (0.830)
SCV beyond 12 months	-0.463 (0.453)	0.130 (0.865)	0.130 (0.769)
5 years SCV	0.072 (0.921)	-0.200 (0.572)	0.068 (0.698)
Overall SCV	-0.155 (0.675)	-0.150 (0.442)	-0.110 (0.547)
Recurrent respiratory infections	0.035 (0.961)	-0.120 (0.515)	-0.140 (0.486)
Recurrent GIT	0.144 (0.845)	-0.140 (0.671)	-0.320 (0.324)

Abbreviations: GIT: gastrointestinal tract, HEU: HIV-exposed uninfected, HUU: HIV-unexposed uninfected, LT: long term, MT: medium term, NK: natural killer, rho: Spearman coefficient, SCV: sick clinic visits

## Discussion

Although several studies have shown changes in NK cell populations in young infants born to HIV infected women, analyses of NK cells in older children are lacking [8, 9]. Here, we demonstrated that the main subsets of NK cells in 5-year-old HEU and HUU children are similar, irrespective of the timing of maternal ART initiation. These findings suggest that although maternal HIV infection may impact NK cell populations early in life, these changes may be reversed with age in HEU children. In line with these immunological studies, HIV and ART exposures were also not associated with increased morbidity in exposed uninfected children compared to unexposed children. In summary, these clinical and immunological analyses suggest that a potential NK cell impairment early in life due to ART and HIV may resolve with age.

NK cell functionality depends on an education process that relies on the expression of inhibitory receptors such as NKG2A and KIRs [12]. The frequencies of NKG2A^+^ CD56^dim^ NK cells and panKIR^+^CD56^dim^ NK cells among HEU children were similar to those in HUU children. These observations are consistent with findings from a study by Ballan et al., which showed that the expression of NKG2A was not significantly different between exposed uninfected and infected children [27]. However, when comparing HEU children with early-life ART exposure, HEULT children however, had slightly reduced frequencies of NKG2A^+^CD56^bright^ NK cells compared to HEUMT children. Considering the small differences, the lower frequencies of NKG2A^+^CD56^bright^ NK cells in HEULT children are unlikely to have severe clinical consequences but larger studies are needed to further determine the potential clinical consequences.

Perforin and granzyme B constitute the cytotoxic arsenal of NK cells to kill virus infected cells [28]. Importantly, the frequencies of intracellular perforin and granzyme B double positive NK cells were similar between HEU and HUU children. Previous studies reported lower perforin levels in HEU infants than in HUU infants under one year of age [8, 9] however, at the age of 5 years these differences were not detected in this study. Due to the small volume of blood collected the current study focused on a phenotypic NK cell characterization, however NK cell functionality may still be affected by maternal HIV and ART exposure. To this end functional analyses of NK cells in terms of degranulation as measured by CD107a or cytokine production are needed. The lack of increased childhood morbidity further suggests that NK cell functionality is not severely impaired.

Although small differences between the HUELT and HEUMT groups were observed, the overall proportions of NK cell subsets and their phenotypes were similar among all groups. The evaluation of NK cell parameters in children with different durations of maternal ART exposure provides an approach to study potential associations with ART duration. These findings are reassuring that phenotypic differences in NK cells in the first years of life may resolve as these differences were not observed at 5 years of age in our study.

The collection of clinical and immunological data allowed us to investigate correlations between NK cell subsets and clinical parameters. A decrease in CD56^dim^ NK cells had a moderate correlation with an increase in sick clinic visits and recurrent respiratory infections in HUU but not in HEU children. These observations may indicate an association between decreased frequencies of CD56^dim^ NK cells and increased risk of respiratory infections in general. Similarly, low frequencies of NK cells in circulation have been associated with viral respiratory infections such as respiratory syncytial virus and severe infection with influenza virus [29, 30]. Furthermore, a study in children with recurrent respiratory infections showed dysregulation of the NK cell compartment in these children compared to controls indicating the possible involvement of NK cells in respiratory infections [31, 32]. While NK cells are crucial for combating intracellular infections, their role in extracellular infections is limited. This distinction is important for interpreting our findings on the observed morbidities. Future research should aim to identify the specific pathogens involved in these respiratory infections to enhance our understanding of the interactions between immune cells, including NK cells, and the nature of the infections.

Given that NK cells are involved in the early immune response against viral infections, it is possible that the reduced CD56^dim^ NK cells may be a result of recurrent viral infection-induced depletion of NK cell subsets [33, 34]. However, further studies are needed to investigate whether decreased frequencies of NK cell subsets may underlie increased frequencies of infections HUU children, in particular identifying the cut-off of absolute NK cells in PBMCs may be helpful to for identifying impaired NK cell immunity. However, these studies require simultaneous analyses of other immune cells such as B cells and T cells, to decipher the specific contribution of NK cells as we observed weak correlations with morbidity in the HEU group. In the present study these markers were not available.

### Strengths and limitations

Previously reported NK cell perturbations among HEU children compared to HUU children have been observed in infants younger than one year. The present study presents a phenotypic assessment of NK cells in an older cohort of HEU and HUU children who were matched for age, from the same location, and had relatively similar socioeconomic statuses to avoid any confounders such as environmental or age-related factors. Specific growth and health outcomes among exposed uninfected children have been suggested to differ according to the duration of *in utero* ART exposure [35, 36]. This relatively large study allowed us to further classify HEU children by duration of ART exposure into HEULT and HEUMT children [26] and one of the few studies to correlate the NK subsets with clinical symptoms. The deep immunophenotyping and clinical analyses revealed that NK cell populations and clinical parameters are not associated with maternal ART or HIV infection.

Absence of samples from the same participants at an earlier age restricted our ability to analyse NK cell dynamics during early life. NK cells are essential in protection against intracellular viral and bacterial infections. However, the pathogen causing the respiratory tract infections in these children was not assessed. Due to the limited sample size in the HEI group of children, we were unable to include these analyses in the main study. As a result, findings for this group should be interpreted with caution and may serve as a preliminary basis for future research. Larger studies involving cohorts of HEI children with varying timing of vertical HIV transmission are essential to understand the impact of transmission mode and viremia on their health at five years of age.

## Conclusion

In summary, these findings demonstrate that the proportions of NK cells in HEU children are relatively similar to those in HUU children at the age of five years, suggesting that early differences may be restored after infancy. Furthermore, we identified that lower CD56^dim^ NK cells were associated with recurrent respiratory infections and thus may be further explored as a marker of immune competence.

## Electronic supplementary material

Below is the link to the electronic supplementary material.

Supplementary Material 1

Supplementary Material 2

## Data Availability

The datasets used and analyzed during the current study are available from the corresponding author upon reasonable request.

## Abbreviations

ART: Antiretroviral therapy
CD4: Cluster of differentiation 4
CD57: Cluster of differentiation 57
DNA: Deoxyribonucleic acid
JREC: Joint research ethics committee of the university of zimbabwe and parirenyatwa group hospitals
HEI: HIV exposed and infected
HEU: HIV-exposed but uninfected
HUU: HIV-unexposed uninfected
HIV: Human immunodeficiency virus
KIR: Killer immunoglobulin-like receptor
MTCT: Mother to child transmission
MUAC: Mid-upper arm circumference
MRCZ: Medical research council of zimbabwe
NK: Natural killer
PBMC: Peripheral blood mononuclear cells
RT-PCR: Reverse transcriptase polymerase chain reaction
PLWH: People living with HIV
PMTCT: Prevention of mother to child transmission of HIV
TENOLAM-E: Tenofovir, lamivudine and efavirenz
UNAIDS: The joint united nations programme on hiv/AIDS
UZBCS: University of zimbabwe birth cohort study
UZFMHS: University of zimbabwe faculty of medicine and health sciences
