# Supplementary material for: Phenotypic characterization of NK cells in 5-year-old children exposed to maternal HIV and antiretroviral therapy in early-life
Source: BMC Immunol. 2024 Dec 19;25:82. doi: 10.1186/s12865-024-00674-4 (PMC11658373; doi:10.1186/s12865-024-00674-4)
Supplement: Supplementary file 1 — Supplementary Material 1 [file 12865_2024_674_MOESM1_ESM.pdf]

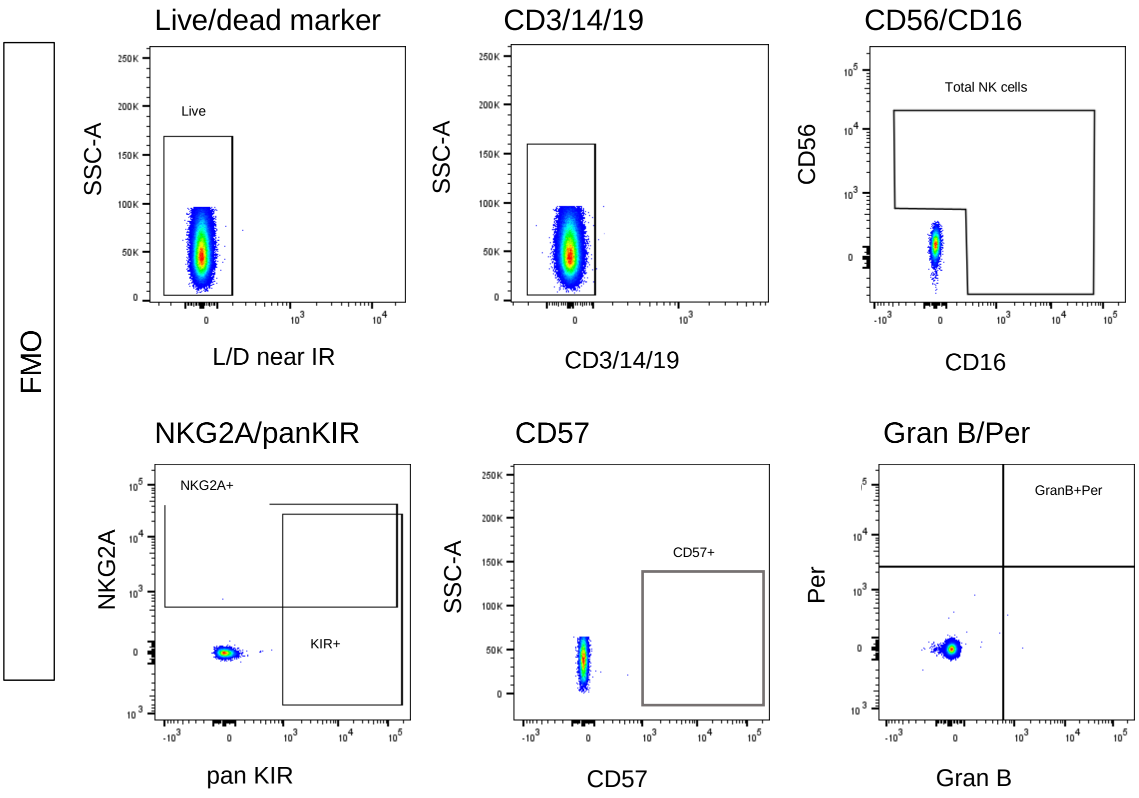

Supplementary Figure 1: Gating of NK cell populations using Fluorescence minus one controls. Abbreviations- FMO: Fluorescence Minus One, GranB: Granzyme B, Per: Perforin, SSC-A: Side scatter area.

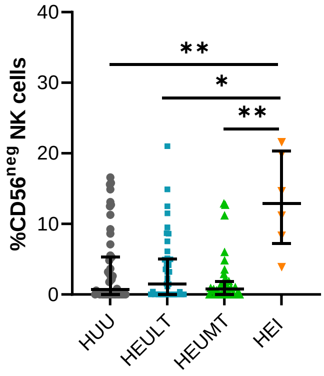

Supplementary Figure 2: Frequencies of CD56^{neg}CD16^{bright} NK cells among HUU, HEUMT, HEULT and HEI children. *=p< 0.05, **=p<0.001. Abbreviations: HEI: HIV-exposed infected, HEU: HIV-exposed uninfected, HUU: HIV-unexposed uninfected, LT: long term, MT: medium term, neg: negative, NK: natural killer.

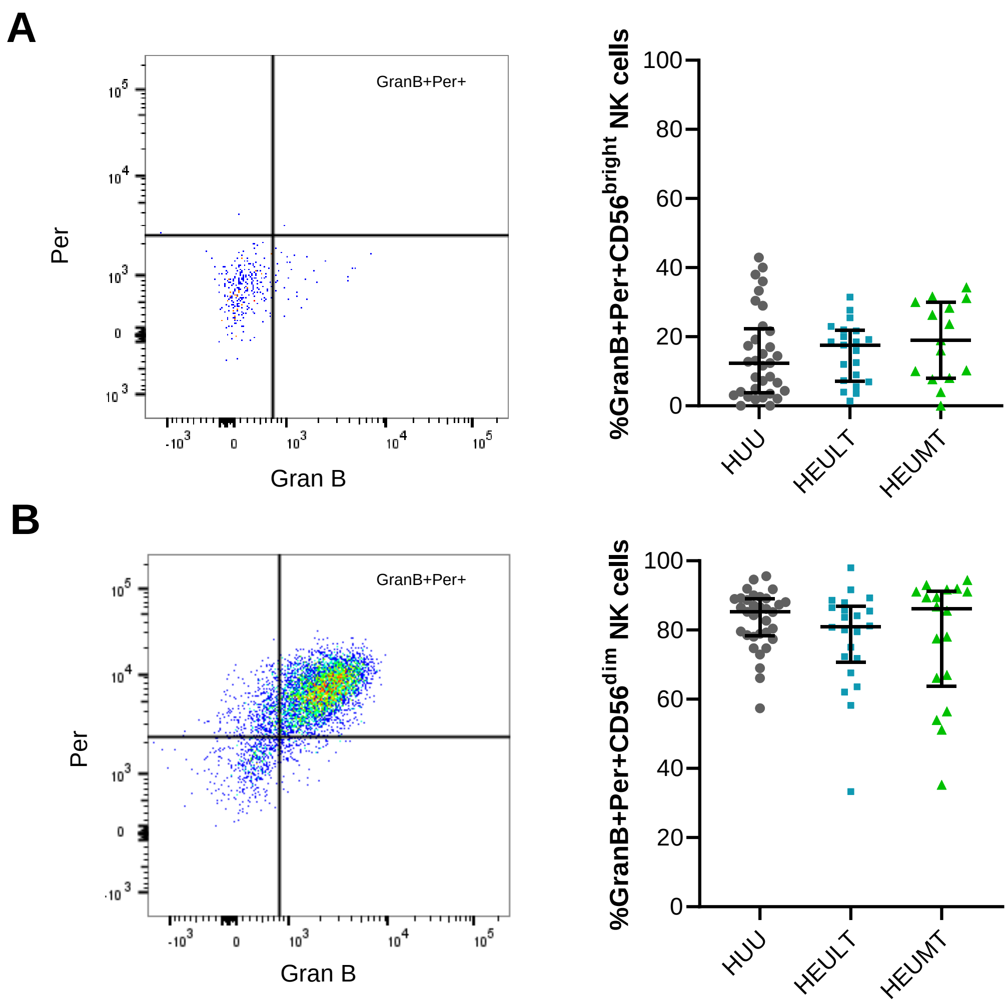

Supplementary Figure 3: Frequencies of (**A**) intracellular granzyme B^{+}perforin^{+} CD56^{bright} NK cells and (**B**) granzyme B^{+}perforin^{+} CD56^{dim} NK cells in HUU, HEUMT and HEULT children. Abbreviations: GranB: Granzyme B, HEU: HIV-exposed uninfected, HUU: HIV-unexposed uninfected, LT: long term, MT: medium term, NK: natural killer, Per: Perforin.

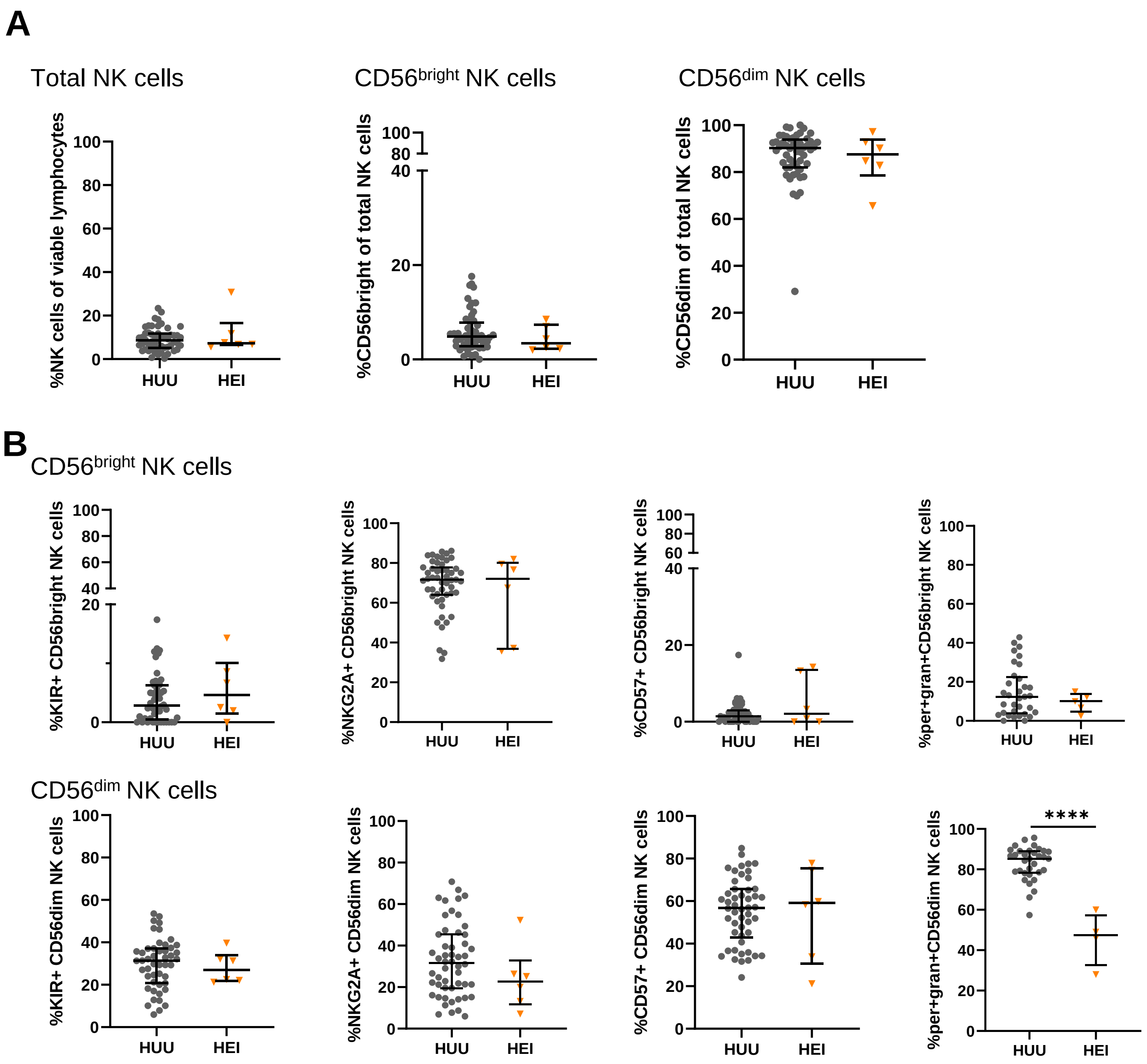

Supplementary Figure 4: (A) The frequencies of total NK cells among viable peripheral blood lymphocytes and subsets (CD56^{bright} and CD56^{dim} NK cells) and (**B**) Summarized data of the percentages of CD56^{bright} and CD56^{dim} NK cells expressing NKG2A and KIR3DL1/KIR2DL2/L3 (pan-KIR), CD57 and intracellular perforin/granzyme B among HUU and HEI children.
