# Supplementary material for: Phenotypic characterization of NK cells in 5-year-old children exposed to maternal HIV and antiretroviral therapy in early-life
Source: BMC Immunol. 2024 Dec 19;25:82. doi: 10.1186/s12865-024-00674-4 (PMC11658373; doi:10.1186/s12865-024-00674-4)
Supplement: Supplementary file 2 — Supplementary Material 2 [file 12865_2024_674_MOESM2_ESM.docx]

**Supplementary Table 1:** Maternal factors in pregnancy and infant birth outcomes stratified by HIV and ART exposure status.

| Characteristic  Median (1^st^ IQR-3^rd^IQR)  [N(%)] | Overall  (N=139) | HEU  (N=81) | | HUU  (N=52) | HEI  (N=6) | P value |
| --- | --- | --- | --- | --- | --- | --- |
|  |  | **HEU LT**  **(N=43)** | **HEU MT (N=38)** |  |  |  |
| Maternal factors at enrolment | | | | | | |
| Age | 30.0(25.5-35.0) | 33.0(29.0-35.5) | 30.0(24.0-35.5) | 29.0(25.0-34.0) | 23.5(19.8-26.5) | **0.014** |
| Viral load (N=87)  Suppressed  Unsuppressed | 72(84.7)  13(15.3) | 39(95.1)  2(4.9)  (missing=2) | 33(86.8)  5(13.2) | N/A | 0  6(100) | **<0.001*** |
| CD4 count (N=87) | 415(264-567) | 422(308-535) | 347(230-589) | N/A | 391(250-496) | 0.679* |
| Infant factors at birth | | | | | | |
| Gestational age at birth (weeks)  Preterm (<37)  Term (≥37) | 12(8.9)  123(91.1)  (missing=4) | 8(19.0)  34(81.0)  (missing=1) | 2(5.7)  33(94.3)  (missing=3) | 2(3.9)  50(96.1) | 0  6(100) | 0.082 |
| Birth weight(grams)  <2500  ≥2500 | 13(9.4)  126(90.6) | 4(9.3)  39(90.7) | 6(15.8)  32(84.2) | 3(5.8)  49(94.2) | 0  6(100) | 0.434 |
| Sex  Female  Male | 67(48.2)  72(51.8) | 23(53.5)  20(46.5) | 19(50.0)  19(50.0) | 23(44.2)  29(55.8) | 2(33.3)  4(66.7) | 0.609 |
| Timing of seroconversion  Peri-partum  Postpartum | 3(50.0)  3(50.0) | N/A | N/A | N/A | 3(50.0)  3(50.0) | - |

*comparison between HEI, HEULT and HEUMT, N/A- not applicable

Abbreviations: HEI: HIV-exposed infected, HEU: HIV-exposed uninfected, HUU: HIV-unexposed uninfected, LT: long term, MT: medium term

**Supplementary Table 2:** Social demographics, morbidity and clinical data for children stratified by HIV and ART exposure status.

| Characteristic  Median (1^st^ IQR-3^rd^ IQR)  [N(%)] | HEU  (N=81) | | HUU  (N=52) | HEI  (N=6) | P value^^^ | P value^#^ |  |
| --- | --- | --- | --- | --- | --- | --- | --- |
|  | **HEU MT (N=38)** | **HEU LT**  **(N=43)** |  |  |  |  |  |
| Social demographic factor at 5 years | | | | | |  |  |
| Age (years) | 5.0 (4.6-5.2) | 5.2(4.8-6.0) | 5.2 (4.7-6.0) | 4.9 (4.8-5.2) | 0.228 | 0.086 |  |
| Morbidity (birth to 5 years) | | | | | |  |  |
| Sick clinic visits at ≤ 6 weeks of age | 1.0(1.0-1.0) | 1.0(1.0-1.0) | 1.0(1.0-1.0) | 1.0(1.0-1.0) | 0.659 | 0.549 | |
| Sick clinic visits at >6 weeks-6months | 1.0(1.0-1.0) | 1.0(1.0-1.0) | 1.0(1.0-1.0) | 1.0(1.0-1.5) | 0.831 | 0.855 | |
| Sick clinic visits at > 6 months-12months | 1.0(1.0-2.0) | 1.0(1.0-1.3) | 1.0(1.0-1.0) | 1.5(1.3-1.8) | 0.723 | 0.596 | |
| Sick clinic visits beyond 12 months | 1.0(1.0-2.0) | 1.0(1.0-2.0) | 1.0(1.0-2.0) | 2.0(1.5-3.0) | 0.756 | 0.974 | |
| Combined sick clinic visit (birth- 5 years) | 2.0(1.0-3.0) | 2.0(1.0-3.0) | 2.0(1.0-3.0) | 2.5(1.3-4.5) | 0.518 | 0.398 | |
| Hospitalization  Yes  No | 3(7.9)  35(92.1) | 4(9.3)  39(90.7) | 6(11.5)  46(88.5) | 1(16.7)  5(83.3) | 0.724 | 0.875 | |
| Clinical data at 5 years | | | | | |  |  |
| Weight (kg) | 17.7(16.0-19.7) | 17.9(15.7-20.0) | 17.7(16.0-19.2) | 17.6(16.2-19.1) | 0.929 | 0.822 | |
| MUAC (cm) | 16.0(15.4-17.0) | 16.4(15.5-17.0) | 16.0(15.5-17.0) | 16.0(15.1-16.9) | 0.911 | 0.798 | |
| HB(g/dL) | 13.5(12.5-14.5) | 13.4(12.7-14.2) | 13.4(12.7-14.1) | 11.9(11.5-13.7) | 0.517 | 0.668 | |
| WBC (10^9^ /L) | 7.0(5.5-8.9) | 6.2(5.2-7.1) | 6.9(5.7-8.6) | 6.0(5.1-7.3) | 0.225 | 0.120 | |
| ART therapy  Yes  No | N/A | N/A | N/A | 5(83.3)  1(16.7) |  | - | |
| ART  Abacavir/ Lamivudine  Dolutegravir  Lopinavir | N/A | N/A | N/A | 6  5  1 |  | - | |
| HIV suppression (copies/ml)  Suppressed (≤1000)  Unsuppressed (>1000) | N/A | N/A | N/A | 3(50.0)  3(50.0) |  | - | |

^- p values for comparisons between HEULT, HEUMT, HEI and HUU, #- p values for comparisons between HEULT, HEUMT and HUU. N/A- not applicable.

Abbreviations; ART: antiretroviral therapy, cm: centimetres, dl: decilitres, HB: haemoglobin, HEI: HIV-exposed infected; HEU: HIV-exposed uninfected; HUU: HIV-unexposed uninfected; kg: kilograms, LT: long term; MT: medium term, MUAC: mid upper-arm circumference, VL: viral load, WBC: white blood cell count
